# Adverse clinical outcomes and associated factors among older adults undergoing hemodialysis in Brazil: A single-center experience

**DOI:** 10.1371/journal.pone.0330030

**Published:** 2025-08-18

**Authors:** Laurisson Albuquerque da Costa, Andre Faro, Thaisa Leite Valverde, Somkanya Tungsanga, Aminu K. Bello

**Affiliations:** 1 Health Psychology Laboratory (GEPPS), Department of Psychology, Federal University of Sergipe, São Cristóvão, Sergipe, Brazil; 2 University Hospital of Sergipe, Federal University of Sergipe, Aracaju, Sergipe, Brazil; 3 Division of Nephrology and Immunology, Department of Medicine, University of Alberta, Faculty of Medicine and Dentistry, Edmonton, Alberta, Canada; 4 Division of General Internal Medicine-Nephrology, Department of Medicine, Faculty of Medicine, Chulalongkorn University, Bangkok, Thailand; Warren Alpert Medical School of Brown University: Brown University Warren Alpert Medical School, UNITED STATES OF AMERICA

## Abstract

**Introduction:**

In the last decade, there has been an increase in the number of older adults diagnosed with kidney failure in Brazil. Anecdotal reports suggest that older adults receiving hemodialysis (HD) face a higher risk of adverse outcomes. This study aims to investigate adverse clinical outcomes and associated factors among adults over age 60 who received chronic HD at a single center in northeastern Brazil.

**Methodology:**

We conducted a retrospective cohort of older adults undergoing HD at a center in Aracaju, Sergipe, Brazil, from October 1, 2019 to March 1, 2024. Multivariable Cox regression analysis was performed to examine the associations with various risk factors for all-cause mortality. A binomial logistic regression model was leveraged for Major Cardiovascular Events (MACE) and all-cause hospitalization.

**Results:**

Among the 950 adults, 392 individuals over age 60 were included in our sample (median age: 68.5 years, IQR: 64–75; male: 63%). Diabetes was the leading cause of kidney failure. The total number of deaths was 157 (40.1%), primarily due to infection (n = 60, 38.2%). Multivariable analysis indicated that increased age was independently associated with all-cause mortality [HR = 1.07 (1.02–1.09), *p* = 0.001], while fistula use was associated with reduced mortality risk [HR = 0.36 (0.19–0.68), *p* = 0.02]. Although hospitalization rate increased with age, this relationship is not statistically significant. Health insurance and hypertension increased hospitalization risk, whereas fistula use was protective. Previous history of cardiovascular disease (CVD) and low serum albumin were associated with MACE. The bloodstream infection rate was 0.18 episode/patient-year, predominantly due to gram-positive organisms, with coagulase-negative *Staphylococcus* being the most common.

**Conclusion:**

Among patients undergoing HD, older age is associated with a high risk of all-cause mortality. Fistula use appeared to be protective against all-cause mortality and hospitalization. Well-designed prospective studies are needed to clarify factors impacting adverse outcomes among older dialysis patients in Brazil.

## Introduction

In recent years, Brazil’s population has aged significantly, with the number of people age 60 and older increasing by 56.0% between 2010 and 2022. By 2021, life expectancy had increased to 77 years, and the Brazilian Institute of Geography and Statistics anticipates that it will reach 81 years by 2060 [1]. Similarly, there has been an increase in the prevalence of kidney failure, particularly among older people, who are initiating hemodialysis (HD) at a faster rate than younger populations [2]. Data from the Brazilian Society of Nephrology show that the number of people undergoing HD increased by 3.6% in 2022 compared to 2021; in 2023, an estimated 157,357 people received dialysis, a prevalence of 771 per million population (pmp) [2]. Between 2015 and 2023, the percentage of people over age 60 initiating dialysis rose from 43% to 48% [3].

The health system in Brazil is composed of the private system (used by individuals with health insurance), philanthropic organizations, and a Unified Health System (UHS) based on the principles of universality, equity and integrity, one of the largest public systems in the world [4]. The private system is supplementary and operates in partnership with UHS, especially for services that cannot be provided by the public system. Therefore, private dialysis units can receive payments from health insurance or UHS [5]. In 2023, 81% of the dialysis centers in Brazil were private, 7% were public and 12% were philanthropic. However, 76% of the individuals on dialysis were financed by UHS [2].

In the Latin American context, a similar trend of increase prevalence of kidney replacement therapy (KRT) is observed. From 1991 to 2013, the prevalence of people requiring KRT increased from 119 pmp to 660 pmp, with a high prevalence among those over age 65, especially in Argentina, Brazil, Chile, Mexico, Puerto Rico, and Uruguay [6]. Data from the Latin American HD and Renal Transplantation Registry show that the prevalence of KRT increased by 28% between 2013 and 2020, reaching 848 pmp [7]. Despite these concerning trends, there is little information regarding outcomes such as all-cause mortality and hospitalization of the elderly population in regions with lower economic indices. Between 2011 and 2017, 82.7% of data from the Brazilian registry for this population came from HD centers located in the south and southeast regions of the country, which have higher economic indices, whereas data from the north and northeast were scarce [8].

It has been well-established that several factors could influence the decision to start HD treatment in older people, such as morbidity, quality of life, symptom relief, life expectancy, and individual preferences. Indeed, elderly patients treated with HD have higher all-cause mortality and hospitalization rates than younger patients, and often have clinical characteristics such as frailty, concerns about vascular access, poor nutrition, cognitive changes, comorbidities, and polypharmacy [9]. These factors make it challenging to improve clinical outcomes for older patients receiving HD [10]. Taking all these issues into consideration, the KDIGO 2024 Clinical Practice Guidelines for Chronic Kidney Disease (CKD) emphasized that care management for this high-risk population must be individualized [11]. Because understanding factors that may be related to unfavorable outcomes for elderly patients is essential to enhance and personalize kidney care, we aimed to investigate adverse clinical outcomes and associated factors among adults over age 60 who received chronic HD at a single center in northeastern Brazil.

## Materials and methods

### Study setting

**Fig 1:**
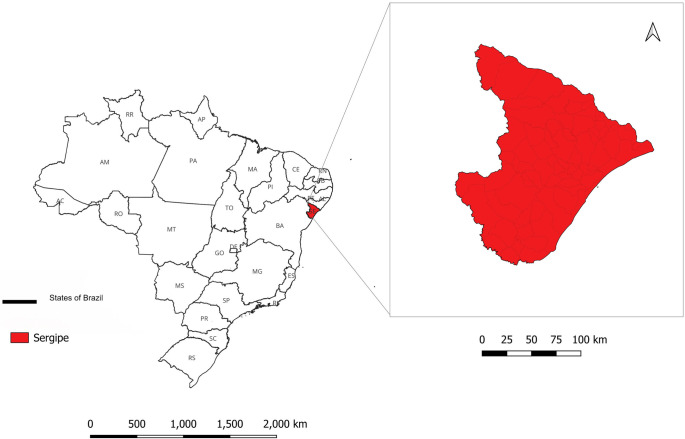
The political and administrative divisions of Brazil, including 26 states and the Federal District. The state of Sergipe is highlighted in red. Map created entirely by the authors using QGIS version 3.36 (https://qgis.org), without the use of any copyrighted or proprietary data. The information presented in Figure 1 was derived from public domain data provided by the Brazilian Institute of Geography and Statistics (IBGE) [1].

According to the Brazilian census of dialysis, in 2023, 19% of dialysis centers were located in the northeast [2]. The dialysis center in this study is situated in Sergipe, the smallest state in Brazil, in the northeast region. The population exceeds 2 million people, with the proportion of older adults increasing from 7.6% in 2000 to 13.3% in 2023. Additionally, life expectancy in the state reached 76.1 years in 2023 (Fig 1) [1].

Nephrology services in Sergipe are organized across five private dialysis centers that provide HD treatment for individuals with CKD, one of which also offers peritoneal dialysis (PD). Participation in the Brazilian dialysis registry is voluntary; in the most recent dialysis census, only one center in Sergipe reported data [2]. Although data are scarce, estimates indicate that 1,592 outpatients were receiving in-center dialysis as of September 2024, with 1,411 (88.6%) receiving HD and 181 (11.4%) receiving PD, and 1,318 (82.8%) paying for dialysis through UHS and 274 (17.2%) using supplementary health insurance. The dialysis center in this study administered HD to 509 individuals (36.1% of HD patients in the state). One public service has been performing living donor kidney transplants since 2022. Importantly, due to the high demand for dialysis, individuals who receive outpatient services must join a waitlist for dialysis services after 2–4 weeks of missed appointments (e.g., due to hospitalization).

### Study design and participants

This is a retrospective cohort study of older individuals undergoing HD at a center in the city of Aracaju, in the state of Sergipe, Brazil between October 1, 2019 and March 1, 2024. Among the 986 individuals who initiated HD during the period, 36 were excluded because they (a) were younger than age 18, or (b) had acute kidney injury and recovered kidney function within 3 months. Among the 950 adult patients who received HD at the center, all 392 individuals over age 60 were included in the study. All patients underwent in-center high-flux HD and none had chronic infections such as HIV or hepatitis B or C. None of the patients had peritoneal dialysis prior HD, and only three had a history of kidney transplantation.

### Data collation

Between March 1, 2024 and July 15, 2024, we collected secondary clinical and laboratory data from the International Renal Information Management System (iRIMS) databases, a platform developed in Sweden to facilitate the management of medical records, patient information, and laboratory data. To ensure confidentiality and anonymity, all data were coded and anonymized. Records from the clinic were accessed only after receiving written authorization from the directors and responsible authorities of the dialysis unit, in accordance with the institution’s policy. This study was conducted in accordance with the Declaration of Helsinki and was approved by the Ethics Advisory Committee of the Federal University of Sergipe (Approval number: 73536223.7.1001.5546).

Clinical characteristics were collected at baseline, including age, gender, etiology of CKD, source of payment (health insurance or UHS), comorbidities (hypertension, diabetes, cardiovascular disease [CVD)] cerebrovascular disease, and cancer), and race (white, black, or mixed-race, a Brazilian description of race used in many studies) [12]. Laboratory measures included serum creatinine, hemoglobin, calcium, phosphorus, transferrin saturation index (TSI), ferritin, intact parathyroid hormone (iPTH), and albumin. The variable infection was defined as the occurrence of at least one documented episode of infection during the study period.

### Study outcomes

Clinical outcomes assessed were all-cause mortality (number of deaths; time to death; and causes, including infection at any site, cardiovascular event, or other), all-cause hospitalizations (frequency; time to first event; and causes, including infection at any site, cardiovascular event, problems related to catheter, or others), major cardiovascular events (including cardiovascular death, non-fatal myocardial infarction, heart failure, and stroke-related hospitalizations), and bloodstream infections related to HD (including incidence density and the pathogen involved). Routine contact was made with patients or families to validate outcomes, specifically to confirm the causes of death or hospitalization. Death certificates were requested to verify deaths, and hospital records were used to confirm details of hospitalizations. Time until death was calculated as the number of days between the first HD session recorded in the databases and the date of death, with hospitalization and infection data tracked starting October 1, 2019.

Reasons for withdrawal from the study included death, kidney transplant, transfer to another center or a hospital, recovery from kidney function, transfer to PD, or HD discontinued by the patient choice.

### Statistical analysis

We used jamovi software (Version 2.5, Sydney, Australia) to analyze the data. Categorical variables are presented as frequency and percentage, and continuous variables are presented as mean ± standard deviation or median (interquartile range), when appropriate. Age was included as a continuous variable to access the incremental effect of age on the adverse outcomes. We used the Shapiro-Wilk test to assess the data for normality of continuous variables. To make comparisons across groups, we used the student’s *t*-test or the Mann-Whitney U-test for quantitative variables, and chi-square calculations for qualitative variables, as appropriate. A *p*-value of < 0.05 was considered statistically significant.

Using logistic regression analysis, we evaluated age as an independent predictor of MACE (including cardiovascular death, non-fatal myocardial infarction, heart failure, or stroke hospitalization) and all-cause hospitalization. We employed multivariable Cox regression modeling to determine independent risk factors associated with all-cause mortality. Variables were included in the models based on statistical analysis and clinical judgment (age, gender, hypertension, diabetes, and CVD).

In the initial bivariate analysis for hospitalization, we included age, health insurance, CVD, fistula usage, albumin, infection, and iPTH because they were statistically significant (*p* < 0.05), as well as gender, hypertension, and diabetes due to their clinical importance. The analysis revealed that the model without infection and iPTH performed the best. In the initial bivariate analysis for MACE, we included the statistically significant variables of health insurance, diabetes, CVD, albumin, and iPTH, as well as age, gender, and hypertension due to clinical judgment. The best model included age, gender, CVD, and albumin. We performed univariate Cox regression analysis to determine the association between each variable and all-cause mortality, and included variables with *p* < 0.15 in the multivariable Cox regression analysis, i.e., age at baseline, hypertension, fistula usage, hemoglobin, TSI, CVD, and albumin, as well as the clinically important variables of gender and diabetes. We performed an additional unadjusted Cox regression analysis to compare all-cause mortality between the adult population (age: 18–60 years) and the older population (age: ≥ 60 years), including two subgroups (age: 60–74 years and ≥ 75 years).

## Results

### Cohort characteristics

Demographic and laboratory data at the beginning of treatment (baseline characteristics) are shown in Table 1. Among 392 older people, the median age was 68.5 years (IQR: 64–75years) and the maximum age was 94 years. Most patients were male (63%) and mixed race (54.3%) and the most common comorbidities were hypertension (87.6%) and diabetes (55.6%). Diabetes was the primary cause of kidney failure in 45.9% of patients, followed by hypertension (24%). A total of 117 patients (30.3%) had a fistula for permanent vascular access, and 194 (50.1%) used private health insurance to pay for HD (Table 1).

**Table 1:** Baseline demographic, clinical and laboratory characteristics (N = 392).

Variable	n (% or IQR)
Age, years	68.5 (64–75)
Male	247 (63)
Comorbidities	
Diabetes mellitus	215 (55.6)
Hypertension	339 (87.6)
CVD	107 (27.6)
Cancer	38 (9.8)
Cerebrovascular disease	34 (8.8)
Peripheral vascular disease	21 (5.4)
Health insurance	194 (50.1)
Etiology of kidney failure	
Diabetes	180 (45.9)
Hypertension	94 (24)
Glomerulonephritis	11 (2.8)
Others	107 (27.3)
Race	
Mixed	213 (54.3)
White	129 (32.9)
Black	50 (12.8)
Use of AV fistula as vascular access	117 (30.3)
Use of CVC	264 (68.4)
Use of graft	5 (1.3)
Hemoglobin, g/dl	8.9 (8.0–10.1)
Serum ferritin, µg/l	347 (166–656)
TSI	19.9 (14–29)
Serum phosphorous, mg/dl	4.6 (3.7–5.4)
Serum calcium, mmol/l	8.6 (8.1–9.2)
Serum iPTH, pg/dl	284 (90.5–359)
Serum creatinine mg/dl	5.8 (4.2–7.0)
Serum albumin, g/dl	3.8 (3.4–4.1)

AV = arteriovenous; CVC = central venous catheter; CVD = cardiovascular disease, defined as heart failure, coronary artery disease or valvular heart disease; IQR = interquartile range; iPTH = intact parathyroid hormone; TSI = transferrin saturation index.

### Clinical outcomes

**Fig 2:**
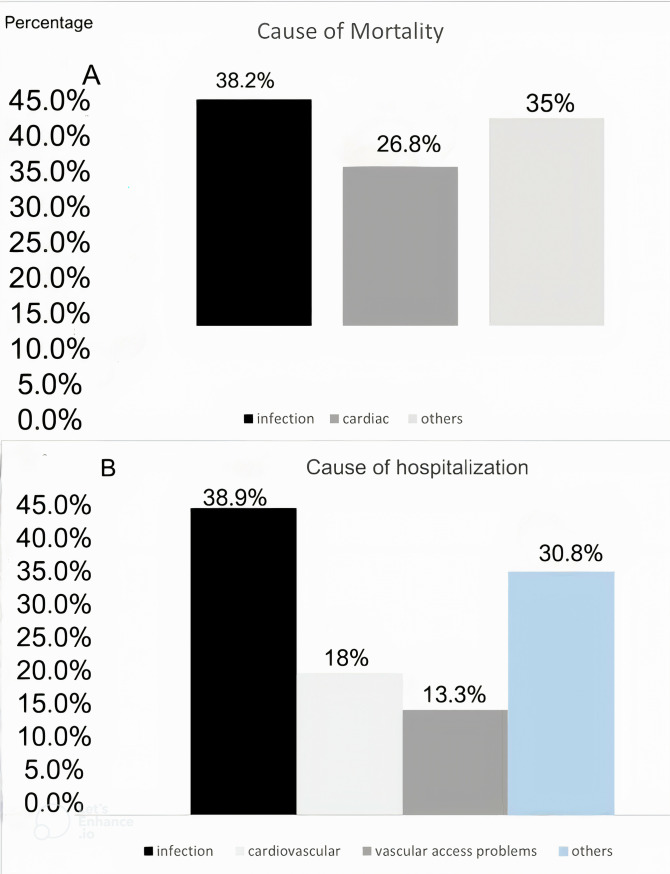
Causes of mortality (A) and hospitalization (B).

**Fig 3:**
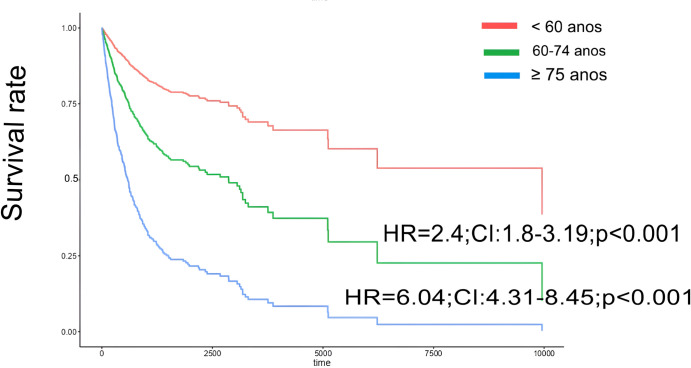
Survival curves for all-cause mortality, stratified by age groups. Comparison between individuals aged <60 years, 60–74 years, and ≥ 75 years.

During the study period, there were 157 deaths (40.1%), with infection as the leading cause (n = 60, 38.2%), primarily related to vascular access; COVID-19 accounted for 10 cases (Fig 2A). Results of the univariate Cox regression analysis indicate that age at baseline, fistula use, serum hemoglobin, and albumin levels predict all-cause mortality (Table 2). Results of the multivariable Cox regression analysis indicate that age is a significant predictor of mortality (HR = 1.07; 95% CI: 1.02, 1.09; **p* *= 0.001), whereas fistula use is associated with a reduced mortality risk (HR = 0.36; 95% CI: 0.19, 0.68; **p* *= 0.02) (Table 3). The risk of death was higher for individuals over age 60 (n = 157, 40.1%) than for those under age 60 (n = 96, 17.2%; HR = 3.05; CI: 2.36, 3.95; *p* < 0.001). Individuals ages 60–74 and 75 or older had 2.4 and 6.04 times the mortality risk, respectively, than those ages 18–59 (Fig 3). The majority (n = 131, 84%) of deaths occurred in the hospital; 19 (12.2%) deaths occurred at home.

**Table 2:** Univariate analyses of the clinical outcomes of all-cause mortality.

Variable	All-cause mortality: Univariate		
	HR	95% CI	*p*
Age	1.07	1.04, 1.10	<.001
Sex (male)	0.86	0.62, 1.18	.341
Hypertension	0.68	0.45, 1.04	.077
Diabetes	0.98	0.71, 1,35	.915
CVD	1.29	0.92, 1.81	.134
Cerebrovascular disease	0.75	0.41, 1.35	.337
Cancer	1.34	0.81, 2.22	.258
Health insurance	1.13	0.82, 1.57	.444
Fistula	0.34	0.23, 0.51	<.001
Mixed-race	1.19	0.87, 1.64	.282
Hemoglobin	0.87	0.78, 0.98	.020
Ferritin	1.00	1.00, 1.00	.784
TSI	0.98	0.96, 1.00	.097
iPTH	1.00	1.00, 1.00	.564
Serum creatinine	0.96	0.89, 1.05	.380
Serum calcium	0.87	0.70, 1.06	.170
Serum phosphorus	0.92	0.79, 1.07	.279
Serum albumin	0.51	0.34, 0.77	.001
Infection	1.65	1.19, 2.28	.003

CI = confidence interval; CVD = cardiovascular disease, defined as heart failure, coronary artery disease or valvular heart disease; HR = hazard ratio; iPTH = intact parathyroid hormone; TSI = transferrin saturation index; **p* *< 0.05 indicates statistical significance.

**Table 3:** Multivariate analyses of the clinical outcomes of all-cause mortality.

Variable	All-cause mortality: Multivariate		
	HR	CI	*p*
Age	1.06	1.02, 1.09	.001
Sex (male)	1.08	0.67, 1.75	.740
Hypertension	0.80	0.39, 1.66	.552
Diabetes	0.74	0.45, 1,23	.244
CVD	0.93	0.54, 1.60	.790
Fistula	0.36	0.19, 0.68	.002
Hemoglobin	0.88	0.77, 1.00	.056
TSI	0.99	0.97, 1.01	.270
Albumin	0.69	0.44, 1.08	.101
Infection	1.38	0.83, 2.28	.211

CI = confidence interval; CVD = cardiovascular disease, defined as heart failure, coronary artery disease or valvular heart disease; HR = hazard ratio; TSI = transferrin saturation index. **p* *< 0.05 indicates statistical significance.

Hospitalization was recorded for 221 individuals (56.37%), each of whom experienced 1–10 episodes, resulting in 483 episodes over the study period, at a rate of 0.52 episodes per patient-year. Infection was the leading cause of hospitalization (Fig 2B). The likelihood of hospitalization increased with age, affecting 51.1% of individuals aged 60–69 years, 63.4% of those aged 70–79 years, and 64.7% of those aged 80 + years. Logistic regression analysis shows that health insurance coverage and hypertension were associated with increased risk of hospitalization, whereas fistula use was associated with a reduced risk. However, age itself was not significantly associated with hospitalization (Table 4).

**Table 4:** Logistic regression of all-cause hospitalization and MACE.

Variable	OR	95% CI	*p*
All-cause hospitalization			
Age (at baseline)	1.01	0.97, 1.05	.562
Sex (male)	0.75	0.44,1.29	.297
Hypertension	2.84	1.16, 6.98	.022
Diabetes	1.08	0.61, 1.89	.799
CVD	1.55	0.81, 2.99	.187
Fistula	0.44	0.24, 0.80	.008
Health insurance	3.00	1.73, 5.22	<.001
Albumin	0.67	0.39, 1.16	.152
MACE			
Age (at baseline)	1.00	0.96, 1.04	.930
Sex (male)	0.81	0.43, 1.52	.510
CVD	3.59	1.87, 6.84	<.001
Albumin	0.49	0.26, 0.92	.026

CI = confidence interval; CVD = cardiovascular disease, defined as heart failure, coronary artery disease or valvular heart disease; MACE = major cardiovascular event; OR = odds ratio; TSI = transferrin saturation index. **p* *< 0.05 indicates statistical significance.

With regard to other adverse outcomes, 90 individuals (23%) experienced major adverse cardiovascular events (MACE). Logistic regression results show that a history of pre-existing CVD and low serum albumin levels are associated with MACE (Table 4).

The incidence rate for blood infections was 0.18 episodes per patient-year, with gram-positive bacteria being the most common causative organisms, notably *Staphylococcus* coagulase negative species (Table 5).

**Table 5:** Types of bacterial bloodstream infection episodes among the study population (n = 167).

Microorganism	n (%)
Gram positive organisms	94 (56.29)
* Staphylococcus aureus*	24 (14.37)
Coagulase-negative *Staphylococci*	56 (33.53)
Other	14 (8.38)
Gram negative organisms	69 (41.38)
* Pseudomonas spp.*	11 (6.59)
Other	158 (34.73)
Fungal organisms	3 (1.80)
*Mycobacterium sp.*	1 (0.60)

Among older patients who discontinued HD at the clinic, only one underwent kidney transplant (0.26%). Other reasons for discontinuation included voluntary cessation (n = 1, 0.26%), switching to PD (n = 18, 4.59%), kidney function recovery (n = 10, 2.55%) after a mean duration of 11.47 ± 7.87 months, transferring to a hospital (n = 20, 5.1%) and moving to a different HD unit (n = 27, 6.89%).

## Discussion

In a cohort of older adults receiving HD at a single center in Brazil, 40.1% experienced mortality, with infection as the leading cause. Our analysis shows that older age at dialysis initiation is linked to a higher all-cause mortality risk, whereas fistula use is linked to a lower risk. Hospitalization occurred in 56.4% of patients, predominantly due to infection, with higher rates observed in older age groups. Health insurance and hypertension are associated with an increased risk of hospitalization, whereas fistula use is associated with a decreased risk. MACE occurred in 23% of patients, with pre-existing CVD and low albumin associated with higher risk. The bloodstream infection rate was 0.18 episodes per patient-year, primarily involving gram-positive bacteria.

Prior research revealed that outcomes for older adults on HD vary across countries and regions [13] and identified several factors linked with unfavorable outcomes. One factor is the type of vascular access; in particular, the use of central venous catheters (CVCs) is related to adverse outcomes such as mortality and hospitalization [14]. In our study, the leading cause of all-cause mortality and hospitalization was infection, with catheter-related infections making a considerable contribution. The low number of individuals with arteriovenous fistulas in this study is consistent with findings based on a Brazilian census of HD patients which show that elderly individuals are 35% more likely to use CVCs than younger individuals, and those receiving treatment in the northeast region of the country are 3.8 times more likely to use CVCs; moreover, similar to our study, the findings show that elderly individuals have a lower survival rate than young people [8]. Our findings make several contributions to the literature.

First, a minority of patients in our sample had fistulas, even though permanent vascular access is associated with better outcomes, such as decreased all-cause mortality and hospitalization [15,16]. It is important to highlight that in many places in Brazil, HD is initiated in emergency situations with temporary non-tunneled catheters; a key factor associated with low dialysis quality indicators in Brazil is the use of a temporary catheter for more than three months [17]. In older adults, decisions regarding vascular access must take into account factors such as life expectancy, comorbidities, patient preferences, vascular history, and quality of life [18]. Although life expectancy must be considered, having a fistula is associated with a lower risk of mortality, even among older people [14].

Second, having private health insurance was associated with an increased risk of hospitalization in our study. This can be attributed to the fact that people with private health insurance have greater access to healthcare facilities, since they can use both public and private systems [19]. Nevertheless, this did not reduce all-cause mortality. In Brazil, access to KRT, expensive medications for anemia and CKD-mineral and bone disorders, as well as emergency services, is universal, with all associated costs fully covered by the UHS. Data from the UHS reveal that in cases of medium and high complexity, KRT accounted for more than 5% of expenses between 2013 and 2015 [20].

Third, in our analysis, the density of hospitalization was 0.52 episode/person-year, most commonly due to infection. In contrast, data from the USRDS show that in 2021, the main cause of hospitalization for individuals ages 65–74 and over age 75 was CVD, with adjusted hospitalization rates of 0.4 and 0.39 admissions per person-year, respectively [21]. For the older population, hospitalization can compromise quality of life and may lead to increased morbidity and all-cause mortality [22,23]. According to the Latin American Registry, 11–20% of patients had at least one episode of hospitalization in their first year of HD during the study period, primarily due to issues with HD catheters (i.e., infection or malfunction) [7]. Our analysis also shows that infection was the main cause of mortality and hospitalization during the study period, even when excluding COVID-19 infections. This finding diverges from high income countries, where CVD typically is the main cause of adverse outcomes among HD recipients [21,24]. Similar to our findings, infection has been shown to be the main cause of all-cause mortality in studies of single-center experiences in middle- to low-income countries [25,26].

Fourth, our findings show a relationship between albumin level and MACE, which may be related to nutritional status. Lower albumin levels and body mass index are associated with an increase in adverse outcomes such as cardiovascular and cerebrovascular disease, hospitalization, and infection [27–30]. Similarly, among individuals over age 80, an analysis of laboratory tests shows a relationship between higher albumin levels and a reduction in cardiovascular and cerebrovascular events in a Korean population [31]. Low albumin is associated with frailty in individuals with CKD [32,33]; older adults also have a higher risk of frailty, which is associated with poor quality of life, risk of hospitalization, infection, CVD events, HD related complications, and mortality [34].

Fifth, although Brazil ranks fourth in transplant activity globally based on the absolute number of kidney transplants performed, only one elderly patient received a kidney transplant during the study period. This may be attributed to concerns about multiple comorbidities, short life expectancy, and the fact that only a small proportion of elderly patients in our study were registered on the transplant waiting list [35], potentially due to a reluctance to travel to other states in Brazil for the transplant procedure.

The number of patients that died in hospitals and the few voluntary discontinuations of HD reflect a need to increase discussions about conservative care, especially for the most elderly patients. Findings are mixed as to whether HD yields beneficial outcomes for elderly patients compared to conservative care, and it may lead to a reduction in quality of life [36].

Although this study has yielded important findings with the capacity to improve clinical outcomes for the elderly population, especially in places with limited resources, it has some limitations, as it is based on a retrospective analysis of data collected from a single center. Furthermore, in the additional comparison between older adults and those under 60 years old, no analysis with adjusted factors was conducted, therefore, the influence of fistula use in this additional analysis could not be evaluated. For individuals with kidney failure, HD and advanced age present complexities that pose significant challenges in patient management and treatment planning. Our findings may support strategies to inform the development of optimal care for patients initiating HD in the state of Sergipe and nationally in Brazil. Care policies that promote vascular access, vaccination, nutrition programs, practices to prevent infection, and discussions about conservative care and withdrawal from HD are needed, along with an increase in the number of kidney transplants for the elderly population.

## Conclusion

Our findings show that for individuals receiving HD, older age is associated with high risks of adverse clinical outcomes. In our study, infections were the leading cause of mortality and hospitalization, whereas fistula appeared to be protective. Well-designed prospective studies are needed to clarify factors impacting adverse outcomes among older individuals who are receiving HD in Brazil. These results provide an important opportunity to devise strategies to mitigate the occurrence of adverse outcomes, thereby improving clinical outcomes and quality of life, and to reduce health disparities for this high-risk population group.
